# High-Intensity Inspiratory Muscle Training Improves Scalene and Sternocleidomastoid Muscle Oxygenation Parameters in Patients With Weaning Difficulties: A Randomized Controlled Trial

**DOI:** 10.3389/fphys.2022.786575

**Published:** 2022-02-09

**Authors:** Marine Van Hollebeke, Diego Poddighe, Beatrix Clerckx, Jan Muller, Greet Hermans, Rik Gosselink, Daniel Langer, Zafeiris Louvaris

**Affiliations:** ^1^Faculty of Movement and Rehabilitation Sciences, Department of Rehabilitation Sciences, Research Group for Rehabilitation in Internal Disorders, KU Leuven, Leuven, Belgium; ^2^Department of Intensive Care Medicine, University Hospitals Leuven, Leuven, Belgium; ^3^Laboratory of Intensive Care Medicine, Department of Cellular and Molecular Medicine, KU Leuven, Leuven, Belgium

**Keywords:** intensive care unit, ventilator weaning, respiratory muscles, metabolism, near-infrared spectroscopy, respiratory muscle training, cell respiration

## Abstract

**Background:**

Critically ill patients who have difficulties weaning from the mechanical ventilator are prone to develop respiratory muscle weakness. Inspiratory muscle training (IMT) can improve respiratory muscle strength. Whether IMT can improve scalene and sternocleidomastoid muscle oxygenation parameters is unknown.

**Aim:**

To compare changes in muscle oxygenation parameters of scalene and sternocleidomastoid inspiratory muscles during a standardized task between patients with weaning difficulties who received either high-intensity IMT (intervention) or sham low-intensity IMT (control).

**Method:**

Forty-one patients performed daily IMT sessions (4 sets, 6–10 breaths) until weaning success or for 28 consecutive days. The training load was progressively adjusted in the intervention group (*n* = 22) to the highest tolerable load, whilst the control group (*n* = 19) kept training at 10% of their baseline maximal inspiratory pressure (PImax). Breathing characteristics (i.e., work and power of breathing, PoB), respiratory muscle function [i.e., PImax and forced vital capacity (FVC)] were measured during a standardized loaded breathing task against a load of 30% of baseline PImax before and after the IMT period. In addition, during the same loaded breathing task, absolute mean and nadir changes from baseline in local scalene and sternocleidomastoid muscle oxygen saturation index (Δ%StiO_2_) (an index of oxygen extraction) and nadir Δ%StiO_2_ normalized for the PoB were measured by near-infrared spectroscopy.

**Results:**

At post measures, only the intervention group improved mean PoB compared to pre measures (Pre: 0.42 ± 0.33 watts, Post: 0.63 ± 0.51watts, *p*-value < 0.01). At post measures, both groups significantly improved nadir scalene muscles StiO_2_% normalized for the mean PoB (ΔStiO_*nadir*_%/watt) compared to pre measurements and the improvement was not significant different between groups (*p*-value = 0.40). However, at post measures, nadir sternocleidomastoid muscle StiO_2_% normalized for the mean PoB (ΔStiO_*nadir*_%/watt) was significantly greater improved in the intervention group (mean difference: +18.4, 95%CI: −1.4; 38.1) compared to the control group (mean difference: +3.7, 95%CI: −18.7; 26.0, between group *p*-value < 0.01). Both groups significantly improved PImax (Intervention: +15 ± 13 cmH_2_O *p*-value < 0.01, Control: +13 ± 15 cmH_2_O *p*-value < 0.01). FVC only significantly improved in the intervention group (+0.33 ± 0.31 L *p* < 0.01) report also change in control group.

**Conclusion:**

This exploratory study suggests that high-intensity IMT induces greater improvements in scalene and sternocleidomastoid muscle oxygenation parameters attributed for oxygen delivery, utilization and oxygen saturation index compared to low-intensity IMT in patients with weaning difficulties.

## Introduction

Approximately 20% of mechanically ventilated patients admitted to the intensive care unit (ICU) experience difficulties with weaning from the mechanical ventilation (Beduneau et al., 2017; Jeong et al., 2018). Patients requiring prolonged invasive mechanical ventilation are prone to develop respiratory and locomotor muscle weakness and have an increased risk of infection, complications, and mortality (Dres et al., 2019; Piva et al., 2019; Sklar et al., 2020; Burns et al., 2021).

Among the multiple causes of weaning failure (Heunks and van der Hoeven, 2010; Perren and Brochard, 2013; Doorduin et al., 2016), respiratory pump failure is considered one of the most common causes (Vassilakopoulos et al., 1996; McConville and Kress, 2012). Pronounced diaphragm weakness, has been observed in more than 60% of mechanically ventilated patients (Dres et al., 2017b). ICU-acquired diaphragm weakness can be apparent within several hours after initiation of ventilator support leading to rapid-onset atrophy and decreased diaphragm contractile force of up to 50% within a few days (Goligher et al., 2015; Berger et al., 2016; Dres et al., 2017b). ICU-acquired diaphragmatic dysfunction is strongly associated with a delayed extubation, prolonged weaning period and weaning failure (de Jonghe et al., 2009; Dres et al., 2017a).

Diaphragm biopsies of mechanically ventilated patients have demonstrated increased proteolysis and decreased protein synthesis, enhanced number of inflammatory cells and loss of muscle mass as important underlying mechanisms to explain reduced force output of the diaphragm (Vassilakopoulos and Petrof, 2004; Hooijman et al., 2015; Berger et al., 2016). Importantly, these alternations do not exclusively affect the diaphragm but, although less rapid, may also affect the other inspiratory and expiratory muscles (Bernard et al., 2003; Capdevila et al., 2003; Shi et al., 2021). Specifically, studies in animal models have found that after 48 h on mechanical ventilation external intercostal muscle of rabbits demonstrated contractile dysfunction and reduction in cross-sectional area of type II fibers (Bernard et al., 2003; Capdevila et al., 2003). A recent study in humans demonstrated time-dependent decreases in abdominal wall muscle thickness due to muscle mass loss in approximately 25% of mechanically ventilated patients (Shi et al., 2021). Furthermore, the increased reliance on the neck inspiratory muscles to overcome the increased respiratory demands has been described in patients with respiratory diseases (De Troyer and Boriek, 2011). In addition, patients who fail a weaning trial, compared to those who wean successfully, present greater electromyographic (EMG) activity of the scalene and sternocleidomastoid muscles that reached near maximal levels within the first 4 min of the SBT trial (Parthasarathy et al., 2007; De Troyer and Boriek, 2011). Weakness of these inspiratory muscles may compromise spontaneous breathing (Parthasarathy et al., 2007).

In this context, inspiratory muscle training (IMT) has been proposed as a strategy to improve respiratory muscle function in ICU patients (Schellekens et al., 2016). In two systematic reviews (Elkins and Dentice, 2015; Vorona et al., 2018) it was observed that IMT is feasible and well-tolerated in critically ill patients and can improve both inspiratory and expiratory muscle strength. IMT might be also beneficial to improve clinical outcomes such as increasing weaning success and reducing the weaning duration (Elkins and Dentice, 2015; Vorona et al., 2018). Studies evaluating other physiological variables beyond assessment of respiratory muscle strength to support the use of IMT in mechanically ventilated patients, have not been performed so far. Training-induced physiological adaptations in local skeletal muscles, oxidative metabolism can be evaluated with near-infrared spectroscopy (NIRS) (Hamaoka et al., 2011; Jones and Cooper, 2014; Grassi and Quaresima, 2016; Barberan-Garcia et al., 2019; Cornelis et al., 2021). Along these lines, IMT has been shown to improved intercostal muscles NIRS-derived oxygenation parameters in athletes and patients with chronic heart failure (Turner et al., 2016; Moreno et al., 2017; Archiza et al., 2018). The effect of IMT on respiratory muscle oxygenation parameters in patients with weaning difficulties has not yet been investigated. Accordingly, the present exploratory study compared changes in scalene and sternocleidomastoid muscle oxygenation parameters in patients with weaning difficulties. Due to the inability to assess oxygenation parameters of diaphragm we focused on scalene and sternocleidomastoid muscles. In addition, key respiratory function variables such as lung volumes, respiratory pressures, work and power of breathing were assessed during a standardized loaded breathing task that was performed before and after the IMT period. We hypothesized that scalene and sternocleidomastoid muscle oxygen saturation index will improve more in response to high-intensity IMT compared to sham low-intensity IMT.

## Materials and Methods

### Study Participants

Patients were recruited from a larger-ongoing Randomized Controlled Trial (RCT) that aims to evaluate the effects of a novel IMT method on weaning outcomes in selected patients with weaning difficulties (clinicaltrials.gov identifier: NCT03240263) (Hoffman et al., 2018). Consequently, the inclusion criteria for this analysis were the same as in the above-cited RCT. All mechanically ventilated patients admitted to the medical and surgical ICU’s between 28th of September 2018 and 10th of November 2020 were screened for eligibility. Patients were eligible if they were not successfully weaned within 24 h after the first separation attempt (Hoffman et al., 2018). A separation attempt was defined under the recently developed classification system for weaning outcomes (Beduneau et al., 2017). Patients were eligible when they met all “readiness to wean” criteria, unable to be weaned within 24 h after the first failed separation attempt, and able to follow simple verbal commands necessary to perform the IMT. Exclusion criteria are described in detail in the published study protocol (Hoffman et al., 2018). Details on eligibility were described in the published study protocol (Hoffman et al., 2018). Written informed consent was obtained from the patient if awake and alert or from a family member. Ethical approval was obtained from the responsible local ethics committee (Ethische Commissie Onderzoek UZ/KU Leuven protocol ID: S60516) and the study has been registered in a publicly accessible clinical trial database (clinicaltrials.gov identifier: NCT03240263). All procedures were performed in accordance with the ethical standards of the ethics committee and with the 1964 Helsinki declaration and its later amendments.

### Study Design and Randomization

This study was designed as a parallel-group, randomized controlled superiority trial with 1:1 allocation ratio. Patients were randomized via block randomization (blocks of 4 and 6), in the intervention group (high-intensity IMT) or the control group (sham low-intensity IMT). Groups were also stratified based on the presence of chronic obstructive pulmonary disease (COPD) and the Acute Physiology and Chronic Health Evaluation II Scale (APACHE II, cut-off: 18) (Hoffman et al., 2018).

### Experimental Procedures

**FIGURE 1:**
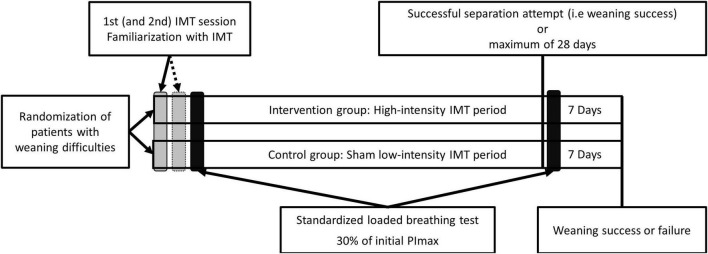
Experimental design. After randomization patients were familiarized during one or two training sessions with the high-intensity inspiratory muscle training (IMT) or sham low-intensity IMT training protocol, in respect to the allocated training group. The subsequent day and the day after the successful separation attempt or after 28 days after inclusion, the patients in both groups performed a standardized loaded breathing task with an external load of approximately 30% of the maximal inspiratory pressure (PImax) of the patient at baseline. IMT was continued until weaning success or failure.

Following randomization, patients in both groups (i.e., intervention and control) participated in an IMT intervention (either sham low-intensity IMT or high-intensity IMT). IMT was continued until the patient was successfully weaned or failed to wean from mechanical ventilation within 28 days in both groups. The clinical team caring for the patient, outcome assessors and patients were blinded for group allocation. Only physiotherapists providing IMT to the patients could not be blinded. In the beginning (after familiarization with IMT, after 1 or 2 IMT sessions) and following the IMT period (after the first successful separation attempt or after 28 weekdays) patients performed a standardized loaded breathing task. During the loaded breathing task, changes in inspiratory muscle oxygenation parameters and breathing characteristics namely inspiratory pressure, inspiratory flow, tidal volume, work of breathing (WoB in Joules) and power of breathing (PoB in watts) were evaluated. The experimental design is presented in Figure 1.

#### Inspiratory Muscle Training

The IMT protocol has been described in detail in the published study protocol (Hoffman et al., 2018). IMT was performed with an electronic tapered flow resistive loading device (POWERbreathe KH2, POWERbreathe International Ltd., Southam, United Kingdom). Visual feedback was available on the laptop screen, which provided information on breathing characteristics (tidal volume, inspiratory flow, WoB, and power) during IMT (Breathe-Link software, version 3.3.2a, POWERbreathe International Ltd., Southam, United Kingdom). Training sessions consisted of approximately 32 breaths consisting of four sets of six to eight breaths per set with a resting period of at least 2 min between the sets. Patients in the intervention group initiated the training with a load corresponding to approximately 30% of their maximal inspiratory pressure (PImax). The load was adjusted daily to the highest tolerable load (corresponding to 30–50% of their PImax) to maximize the WoB and PoB performed during the training. Patients in the control group performed the training against an external load of a maximum of 10% of their PImax. The load was not adjusted during the training period. The training sessions were interrupted if patients reported intolerable symptoms of dyspnoea or discomfort, when systemic arterial oxygen saturation fell below 85% or when the patient started to cough (Hoffman et al., 2018).

### Standardized Loaded Breathing Task

During the standardized loaded breathing task performed at the beginning (after at least 1 training session) and following the IMT period (after the first successful separation attempt or after 28 weekdays, Figure 1), patients were placed in a semi upright position. At rest and during the loaded breathing task, the external load, the number of sets performed, the number of breaths per set, the tidal volume, the time between the sets and the fraction of inspired O_2_ were recorded for each patient and was identically reproduced (except for tidal volumes) at the beginning and following the IMT period. IMT (consisting of four sets of six to eight breaths per set) was performed in both groups with the tapered flow resistive loading device at an inspiratory load of approximately 30% of the patients initial PImax (PImax assessed at baseline). Patients of both groups were encouraged during the loaded breathing tasks to perform maximal, fast and deep inspirations against the inspiratory load and to achieve a full expiration at every breath. Breathing characteristics such as inspiratory pressure, inspiratory flow, tidal volume, WoB and PoB as response to the loaded breathing were recorded for each breath (Breathe-Link software, version 3.3.2a, POWERbreathe International Ltd., Southam, United Kingdom) (Van Hollebeke et al., 2021). Before and after each standardized loaded breathing tasks, heart rate, mean arterial blood pressure (MAP), and oxygen saturation (measured by pulse oximetry) were continuously recorded from the routine bedside monitor. After each standardized loaded breathing task, patients were asked to rate their perceived breathing effort, dyspnoea and unpleasantness on a modified Borg CR-10 scale (Borg, 1982).

### Measures of Respiratory Muscle Function

PImax was measured in a semi-upright sitting position in bed with a unidirectional valve attached to the endotracheal tube or tracheostomy. Inspiration was occluded by the valve but expiration was allowed. Patients were encouraged to perform maximal and forceful inspiratory attempts against the closed valve for an uninterrupted period of 25–30 s (Marini et al., 1986). PImax was measured with a handheld manometer (Pocket-Spiro USB/BT 100, M.E.C., Belgium) and performed three times with at least 2 min rest between the measurements. The highest value of the 1-s plateau pressure was considered for analysis. The forced vital capacity (FVC) and peak inspiratory flow (PIF) were measured with a handheld spirometer (Pocket-Spiro USB/BT 100, M.E.C., Belgium). The spirometer was connected to the endotracheal tube or the tracheostomy. After full inspiration, the patient was asked to perform a full and fast expiration until residual volume followed by a maximal and fast inspiration until total lung capacity. The FVC and PIF were collected simultaneously and was performed three times with at least 2 min rest between the measurements. The best maneuver was considered for analysis. PImax, FVC, and PIF measurements were performed weekly.

### Inspiratory Muscle Oxygenation

**FIGURE 2:**
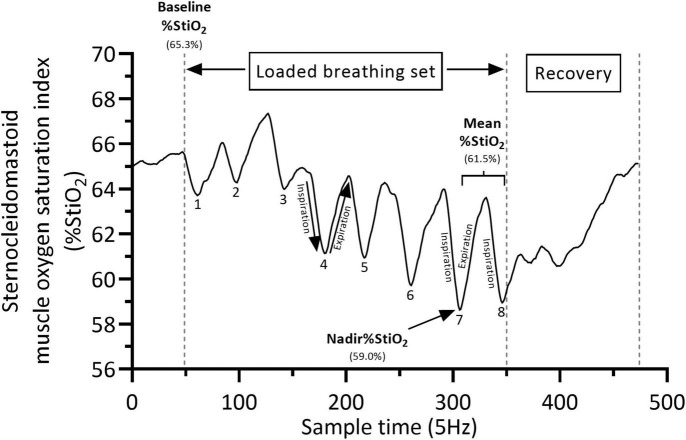
Typical calculation of muscle oxygen saturation index (%StiO_2_) during a loaded breathing set. Baseline %StiO_2_ (defined as the average of the last 10 s before the initiation of each breathing set), Mean %StiO_2_ (defined as the average of the last two breaths of each set) and Nadir %StiO_2_ (defined as the lowest value recorded during the last two breaths of each set). Numbers (from 1 to 8) represent the number of breaths of the set. %StiO_2_ data depict the sternocleidomastoid muscle. %StiO2 changes from baseline are calculated as follow: Mean-Baseline = −3.8% and Nadir– Baseline = −6.3%.

Inspiratory muscle oxygenation parameters were assessed by near-infrared spectroscopy (NIRS, NIRO-200 NX, Hamamatsu, Japan, continuous-wave near-infrared spectroscopy) (Grassi and Quaresima, 2016). NIRS is a continuous, non-invasive method to investigate the changes in oxygenation parameters and hemodynamic responses of muscles and other tissues in real time (Ferrari et al., 2004). Specifically, two sets of NIRS optodes were placed directly on the skin over the scalene muscle (posterior triangle of the neck) and the sternocleidomastoid muscle (halfway along the line from the mastoid bone to the cranial sternal margin) at contralateral sites. NIRS optodes were secured with solid black holders covered in tape to avoid the influence of environmental light on the NIRS signal. Concentration changes in oxy[hemoglobin (Hb) + myoglobin (Mb)] and deoxy (Hb + Mb) in μmol/L, were determined by measuring light attenuation at 760 and 864 nm wavelengths that were analyzed using algorithms based on the modified Beer–Lambert law. Total (Hb + Mb) was calculated as the sum of oxy (Hb + Mb) and deoxy (Hb + Mb) concentrations. Concentration changes in oxy (Hb + Mb) and deoxy (Hb + Mb) were used as indexes of muscles oxygen delivery and oxygen utilization, respectively. Concentration changes in total (Hb + Mb) were used as an index of changes in blood volume reflecting changes in microvascular conductance (vasodilation or vasoconstriction responses) for muscles (Grassi and Quaresima, 2016). In addition, absolute mean and nadir values of NIRS derived muscle oxygen saturation index (%StiO_2_; i.e., the ratio of [oxy (Hb + Mb)] to [total (Hb + Mb) * 100] that reflects the balance between tissue oxygen delivery and oxygen utilization (Grassi and Quaresima, 2016; Barstow, 2019; Louvaris et al., 2021) were also recorded for scalene and sternocleidomastoid muscles. Furthermore, whilst NIRS reflects approximately 70% of venous blood and 30% arterial blood (Boushel et al., 2001), regional %StiO_2_ must reflects regional venous/intracellular oxygenation thus representing an absolute index of muscle oxygen saturation since all regions see the same arterial value (Vogiatzis et al., 2015; Louvaris et al., 2017). The nadir %StiO_2_ values were calculated to allow a better appreciation of the magnitude of scalene and sternocleidomastoid muscles oxygen desaturation during repetitive dynamic contraction of inspiratory muscles in the two groups (Figure 2) (Chuang et al., 2019). To take into account differences in the PoB among patients during pre and post intervention measurements, we normalized Δ%StiO_2nadir_ by the mean PoB during the breathing sets to better appreciate the differences in scalene and sternocleidomastoid muscle oxygen saturation index between the two groups (Barroco et al., 2017; Gephine et al., 2021). Since muscle oxygen saturation level during loaded breathing can be negatively affected by the development of high intramuscular pressures and its effects on microvascular circulation (Rodrigues et al., 2019), nadir %StiO_2_ might also indicate the level of scalene and sternocleidomastoid muscle contraction force produced at pre and post intervention period. NIRS oxygenation data were sampled at 5 Hz. A path length of 18.6 cm was set up for both inspiratory muscles. The separation distance between the NIRS light transmitter and receiver probes was 40 mm, thus allowing a maximum NIRS penetration depth of 20 mm.

All NIRS oxygenation parameters were expressed as changes from baseline. The baseline was defined as the average of the last 10 s before the initiation of each breathing set. During the standardized loaded breathing task, scalene and sternocleidomastoid muscles mean oxygenation values were calculated as the average of the last two breaths of each set and averaged for the 2nd, 3rd, and 4th breathing sets. The 1st set was excluded because the resistance of the first two breaths is automatically set to the lowest load possible (3 cmH_2_O, calibration phase of the device) followed by two breaths with incremental loading (the warming up phase) and truly from the 5th breath the set load was reached. In addition, the nadir %StiO_2_ values for scalene and sternocleidomastoid muscles were defined as the lowest value recorded during the last two breaths of each set and averaged for the 2nd, 3rd, and 4th breathing sets. A representative example for calculating baseline, mean and nadir %StiO_2_ values is shown in Figure 2.

Changes from baseline in nadir scalene and sternocleidomastoid muscle oxygen saturation index (%StiO_2nadir_) were normalized by reporting their values according to the mean PoB during the 2nd, 3rd, and 4th sets and were presented as Δ% StiO_2*nadir*_/watt. Arterial blood gasses were measured daily as part of standard clinical routine. Hemoglobin concentration (Hb) and partial pressure of oxygen were recorded in the morning before the loaded breathing task. Breath by breath data of the patients’ breathing characteristics during the loaded breathing task and during each IMT session were extracted from the Breathe-Link software. For the analysis of the loaded breathing tasks, the breath-by-breath data of the breathing characteristics were disregarded from the first set and average breathing characteristics of the last three sets were calculated.

### Statistical Analysis

Data are expressed as mean ± SD. The primary outcome of the study was the differences in changes in inspiratory muscle oxygen saturation index and normalized muscle oxygen saturation index for the power output before and after the IMT, between the intervention group and the control group. Within-group differences in baseline characteristics and training characteristics (amount of performed training sessions, inspiratory load during the loaded breathing task, and percentage of completed sessions of the planned sessions) were compared with unpaired *t*-tests. Multivariate analysis of variance (MANOVA) was applied to examine whether groups (i.e., control vs. intervention) responded differently to the interventions (high-intensity IMT vs. sham low-intensity IMT) for all the aforementioned variables. *Post hoc* Holm–Šídák multiple comparison tests were applied for within-group comparison for all aforementioned variables. Statistical significance was met when *p* < 0.05. The relationships between potential confounders and primary outcomes were analyzed with Pearson correlation coefficients. These analyses were performed with GraphPad Prism (GraphPad Software, version 9, LCC, United States). Between-group comparisons were corrected for the impact of potential confounders by entering those as covariates with an ANCOVA. These analyses were performed with SPSS Statistics (IBM Corp. Released 2020. IBM SPSS Statistics for Windows, Version 27.0. Armonk, NY, United States: IBM Corp.). As this constitutes the first study to explore the effects of IMT on inspiratory muscle oxygenation parameters in weaning patients, *a priori* sample size calculation was difficult to be performed. Therefore, the minimum sample size required for this study was considered based on observed power that was calculated using the interim data of sternocleidomastoid muscle oxygen saturation index normalized for power output. This analysis included 15 patients in the intervention group and 14 in the control group. By using the formula described by Diggle et al. (2002) and the mean difference in rate of change in the sternocleidomastoid muscle oxygen saturation index normalized for power output between intervention and control group following IMT (i.e., change in control group minus change in intervention group: −4.34 ΔStiO_*nadir*_%/watt) with a power of 80% at a significance level of 5%, the critical sample size was calculated to be 17 patients per training group.

## Results

### Recruitment and Baseline Characteristics

**FIGURE 3:**
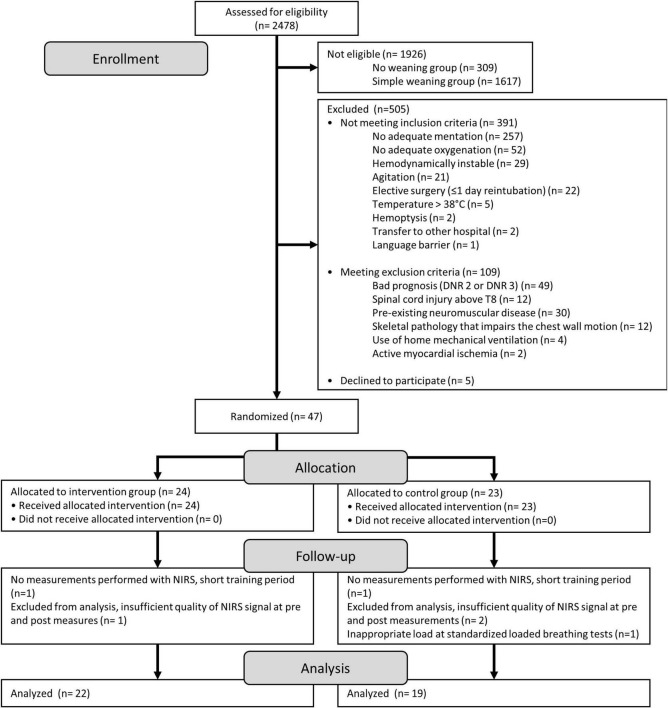
Flowchart of all patients who were screened for eligibility for the study.

A patient flow chart is depicted in Figure 3. Between September 28, 2018 and November 10, 2020, 2,478 mechanically ventilated patients admitted to the surgical and medical ICU were screened for eligibility of which 47 patients were randomized in the study. Of the 47 patients, 6 patients were excluded for analyses (Figure 3). From the remaining 41 patients that were analyzed, 3 patients could not perform the standardized loaded breathing task at the end of the IMT period. One patient in the intervention group (insufficient quality of NIRS signal) and 2 patients in the control group (not adequate at the end of the training period and transferred to another hospital after 1 day of training) had no loaded breathing task at the end of the IMT period. Baseline characteristics of the patients included in the intervention and control group are similar for most of the parameters (Table 1). However, patients randomized in the intervention group were significantly younger and had a significantly lower percent predicted value of FVC compared to patients in the control group (Table 1).

**TABLE 1:** Baseline characteristics.

	Intervention, high-intensity IMT (*n* = 22)	Control, sham low-intensity IMT (*n* = 19)	
			
	Mean *SD*	Mean *SD*	*p*-value
Sex (male/female), *n*	13/9	9/10	0.54
Age, years	52 ± 18	64 ± 7	0.01
Heigth, cm	170 ± 11	170 ± 8	0.91
Weight, kg	72 ± 21	67 ± 17	0.40
BMI	24.7 ± 5.9	23.1 ± 5.1	0.35
Admitted to, *n*			
Surgical ICU	17	17	
Medical ICU	5	2	
Diagnosis, *n*			
Transplantation	14	9	
Lung	11	9	
Lung and kidney	1	0	
Lung and liver	1	0	
Liver and kidney	1	0	
Lung volume reduction surgery	0	1	
Pneumonia	4	2	
Cardiac failure	2	3	
Hematologic	1	0	
Esophageal surgery	0	1	
Maxillofacial surgery	1	0	
Polytrauma	0	1	
Organophosphate intoxication	0	1	
COPD, *n*	3	4	0.68
APACHE-II score, /60	19 ± 8	20 ± 6	0.87
Start IMT, ET-tube/cannula	5/17	1/18	0.19
PImax, cmH_2_O	32 ± 12	35 ± 11	0.67
%pred.	33 ± 13	41 ± 15	0.18
FVC, liter	0.83 ± 0.29	1.00 ± 0.34	0.35
%pred.	22 ± 7	31 ± 12	0.01
Peak inspiratory flow, L/s	0.98 ± 0.29	1.04 ± 0.25	0.57
MV before 1st SA, days	17 ± 24	9 ± 8	0.21
MV before start IMT, days	26 ± 24	27 ± 15	0.87
Ventilator support at inclusion			
Ventilator mode, *n*			
CPAP	16	15	
BIPAP	1	1	
NAVA	4	1	
FiO_2_, %	28 ± 9	31 ± 9	0.25
PEEP, cmH_2_O	6 ± 1	5 ± 1	0.09
PS, cmH_2_O	8 ± 4	7 ± 3	0.60
NAVA level, cmH_2_O/μV	1.0 ± 0.2	3	

*IMT, inspiratory muscle training; BMI, body mass index; ICU, intensive care unit; COPD, chronic obstructive pulmonary disease; APACHE-II score, severity-of-disease classification; IMT, inspiratory muscle training; ET-tube, endotracheal tube; PImax, maximal inspiratory pressure; FVC, forced vital capacity; MV, mechanical ventilation; SA, separation attempt,; CPAP, continuous positive airway pressure; BIPAP, bi-level positive airway pressure; NAVA, neurally adjusted ventilator assist; FiO_2_, fraction of inspired oxygen; PEEP, positive end-expiratory pressure; PS, pressure support; SD, standard deviation. Significance level: p-value < 0.05.*

### Training Data and Progress During Inspiratory Muscle Training

The total amount of training sessions performed and the average completion of planned training sessions was similar between the groups (Table 2). The training load in the intervention group increased by 32% of the patient’s baseline PImax to 57% of the patient’s baseline PImax. The training load in the control group remained under 10% of the PImax and constant throughout the training period (Table 2). Consequently, the total WoB and power of breathing performed during IMT was significantly higher in the intervention group compared to the control group (Table 2).

**TABLE 2:** Training data and breathing characteristics during the first and last inspiratory muscle training session.

	**Intervention group, high-intensity IMT (*n* = 22)**				**Control group, sham low-intensity IMT (*n* = 19)**				**Between group comparison**
			
	**Mean *SD***				**Mean *SD***				***p*-value**
Training days	15 ± 7				15 ± 4				0.79
Completed/planned sessions (%)	74 ± 15				75 ± 15				0.94
Total training volume, joules	274.6 ± 219.5				81.6 ± 45.1				<0.01*

**Breathing characteristics as response to loaded breathing**									

	**First** **session**	**Last** **session**	**Estimated mean difference (last–first)**		**First** **session**	**Last** **session**	**Estimated mean difference (last–first)**		**Multivariate effect**
							
	**Mean *SD***	**Mean *SD***	**Mean**	**95%CI**	**Mean *SD***	**Mean *SD***	**Mean**	**95%CI**	***p*-value**

Training load, cmH_2_O	11.0 ± 4.4	19.4 ± 7.7	8.4	5.5; 11.3*	2.7 ± 0.8	2.7 ± 0.8	0	0; 0	<0.01
Training load, %PImax_*baseline*_	34 ± 5	62 ± 22	29	19; 38*	8 ± 3	8 ± 3	0	0; 0	<0.01
Mean inspiratory flow, L/sec	0.45 ± 0.32	0.50 ± 0.27	0.05	−0.06; 0.15	0.51 ± 0.18	0.52 ± 0.18	0.02	−0.07; 0.11	0.81
Peak inspiratory flow, L/sec	0.92 ± 0.45	1.06 ± 0.45	0.14	−0.03; 0.30	0.87 ± 0.26	0.88 ± 0.27	0.01	−0.11; 0.13	0.29
Tidal volume, L	0.52 ± 0.32	0.65 ± 0.35	0.13	0.00; 0.26*	0.47 ± 0.19	0.68 ± 0.36	0.21	0.08; 0.34*	0.63
Tidal volume, %FVC	61 ± 27	58 ± 30	−3	−18; 12	51 ± 18	67 ± 29	15	−1; 30	0.22
Number of breaths	28 ± 7	26 ± 6	−2	−5; 1	27 ± 5	27 ± 4	0	−3; 2	0.70
Work of breathing/session, joules	14.5 ± 15.1	28.5 ± 24.7	14.1	4.8; 23.3*	5.0 ± 2.6	7.3 ± 4.9	2.3	−8.1; 12.8	<0.01
Mean power of breathing, watts	0.41 ± 0.32	0.78 ± 0.55	0.37	0.19; 0.56*	0.20 ± 0.10	0.20 ± 0.10	0.01	−0.03; 0.04	<0.01
Peak power of breathing, watts	0.98 ± 0.72	2.07 ± 1.42	1.08	0.60; 1.57*	0.35 ± 0.17	0.35 ± 0.16	0.00	−0.05; 0.05	<0.01
Perceived dyspnea, MBS, /10	5 ± 2	4 ± 2	−2	−2; −1*	5 ± 2	3 ± 2	−2	−3; −1*	0.65
Perceived respiratory effort, MBS, /10	6 ± 2	4 ± 2	−1	−2; 0*	5 ± 2	3 ± 1	−2	−3; −1*	0.05
Unpleasantness, VAS, /10	3 ± 3	3 ± 3	0	−1.7; 1.2	4 ± 2	3 ± 2	−1	−2; 1	0.54

Breathing characteristics during the first and the last inspiratory muscle training session are depicted with within and between group comparisons. Last session missing for 1 patient in the control group. IMT, inspiratory muscle training; total training volume, the overall work of breathing performed over the whole course of the inspiratory muscle training period; PImax, maximal inspiratory pressure; FVC, forced vital capacity; %FVC, tidal volume expressed as the percentage of the baseline measurement for the first session and the post FVC measurement for the last session; MBS, modified BORG scale; VAS, visual analog scale; SD, standard deviation; CI, confidence interval. *Significance level: p-value < 0.05.

### Respiratory Muscle Function Before and After Inspiratory Muscle Training

The effects of IMT on respiratory muscle function in patients with weaning difficulties are presented in Table 3. PImax improved in both groups significantly and similarly over the course of the IMT period. Nevertheless, only in the intervention group both FVC expressed in liters and PIF increased significantly over the course of the IMT period. No significant between group differences were observed for FVC expressed in liters and PIF. A significant greater increase in FVC, expressed as percent predicted, was observed in the intervention group compared to the control group (Table 3).

**TABLE 3:** Respiratory muscle function and pulmonary function before (pre) and after (post) IMT.

	Intervention group, high-intensity IMT (*n* = 22)				Control group, sham low-intensity IMT (*n* = 19)				Between group comparison
			
	Pre	Post	Estimated mean difference (post–pre)		Pre	Post	Estimated mean difference (post–pre)		Multivariate effect
	Mean *SD*	Mean *SD*	Mean	95%CI	Mean *SD*	Mean *SD*	Mean	95%CI	*p*-value
Maximal inspiratory pressure, cmH_2_O	32 ± 12	47 ± 15	15	7; 24*	353 ± 11	48 ± 17	13	4; 23*	0.77
Maximal inspiratory pressure, %pred.	32 ± 14	47 ± 15	15	6; 24*	40 ± 15	53 ± 19	14	4; 15*	0.31
Forced vital capacity, L	0.83 ± 0.29	1.16 ± 0.48	0.33	0.07; 0.59*	0.99 ± 0.35	1.09 ± 0.50	0.13	−0.15; 0.41	0.08
Forced vital capacity, %pred	22 ± 7	30 ± 9	8	1; 15*	31 ± 13	32 ± 14	3	−5; 10	0.03
Peak inspiratory flow, L/s	0.98 ± 0.29	1.33 ± 0.48	0.36	0.14; 0.57*	1.04 ± 0.25	1.13 ± 0.29	0.08	−0.16; 0.32	0.06

Respiratory muscle function measured before (pre) and after (post) the inspiratory muscle training period. Post measurement missing for 1 patient in the control group. IMT, inspiratory muscle training; %pred, percent of the predicted value of the maximal inspiratory pressure; SD, standard deviation; CI, confidence interval. *Significance level: p-value < 0.05.

### Breathing Characteristics During the Standardized Loaded Breathing Task

Table 4 presents the breathing characteristics, hemodynamic responses and symptoms during the standardized loaded breathing task. The inspiratory load was comparable between the intervention and control group. At rest, during pre and post measures, both groups presented similar hemodynamic and respiratory parameters (i.e., heart rate, mean arterial pressure, hemoglobin concentration, partial pressure of oxygen, and arterial oxygen saturation). Fraction of inspired O_2_ was also similar during pre and post measures (Supplementary Table 1). In both groups, MAP was significantly decreased at post measurements compared to pre measurements, but no between-group significant differences were observed. Inspiratory flow, tidal volume and WoB per breath were significantly increased in both groups at post measures. The mean and peak PoB were significantly increased only in the intervention group, but no between-group significant differences were observed. In the intervention group, the patients scored the perceived respiratory effort significantly lower at the post measurement compared to the pre-measurement and was not significantly different within the control group. However, no significant between group differences were found in hemodynamic and respiratory responses, breathing characteristics nor perceived symptoms.

**TABLE 4:** Breathing characteristics, hemodynamic responses and symptoms during the sandardized loaded breathing tasks.

	**Intervention group, high-intensity IMT (*n* = 22)**				**Control group, sham low-intensity IMT (*n* = 19)**				**Between group comparison**
			
	**Mean *SD***				**Mean *SD***				***p*-value**
			
Inspiratory load, cmH_2_O	12 ± 5				12 ± 4				0.83
Inspiratory load, % PImax	37 ± 10				34 ± 9				0.30

	**Pre**	**Post**	**Estimated mean difference (post–pre)**		**Pre**	**Post**	**Estimated mean difference (post–pre)**		**Multivariate effects**
							
	**Mean *SD***	**Mean *SD***	**Mean**	**95%CI**	**Mean *SD***	**Mean *SD***	**Mean**	**95%CI**	***p*-value**

**Hemodynamic and respiratory responses to loaded breathing relative to rest**									
**Δ** Heart rate, beats/min	0 ± 15	1 ± 6	1	−6; 9	2 ± 4	2 ± 4	0	−2; 6	0.67
Δ Mean arterial pressure, mmHg	6 ± 7	−2 ± 5	−8	−12; −4*	2 ± 9	−5 ± 10	−7	−15; −1*	0.33
Δ Respiratory rate, breaths/min	1 ± 8	1 ± 7	1	−3; 4	3 ± 5	2 ± 9	0	−5; 5	0.85
Δ Oxygen saturation, %	−1 ± 3	0 ± 3	1	−1; 4	−1 ± 2	−1 ± 3	0	−2; 2	0.58
**Breathing characteristics as response to loaded breathing**									
Mean inspiratory pressure, cmH_2_O	8.4 ± 3.4	8.6 ± 3.0	0.1	−0.6; 0.9	7.8 ± 1.6	7.6 ± 1.8	−0.2	−0.6; 0.3	0.51
Mean inspiratory flow, L/sec	0.40 ± 0.22	0.62 ± 0.32	0.22	0.12; 0.33*	0.35 ± 0.20	0.49 ± 0.14	0.14	0.05; 0.23*	0.29
Peak inspiratory flow, L/sec	0.90 ± 0.42	1.14 ± 0.51	0.23	0.1; 0.38*	0.76 ± 0.36	0.96 ± 0.24	0.19	0.1; 0.33*	0.42
Tidal volume, L	0.49 ± 0.28	0.68 ± 0.31	0.19	0.1; 0.3*	0.39 ± 0.21	0.65 ± 0.30	0.26	0.1; 0.41*	0.47
Tidal volume, %FVC at baseline	59 ± 26	87 ± 44	28	12; 43*	48 ± 30	74 ± 46	26	10; 43*	0.46
Number of breaths	25 ± 9	24 ± 5	−1	−5; 3	23 ± 8	23 ± 7	0	−4; 3	0.75
Total work of breathing, joules	14.3 ± 15.2	16.8 ± 12.9	2.6	−2.0; 7.2	8.3 ± 6.0	14.2 ± 10.5	5.9	1.6; 10.2*	0.30
Work of breathing/breath, joules	0.53 ± 0.45	0.70 ± 0.51	0.17	0.04; 0.30*	0.35 ± 0.22	0.57 ± 0.30	0.22	0.10; 0.35*	0.32
Mean power of breathing, watts	0.42 ± 0.33	0.63 ± 0.51	0.21	0.06; 0.43*	0.31 ± 0.20	0.40 ± 0.17	0.09	−0.14; 0.30	0.24
Peak power of breathing, watts	1.11 ± 0.83	1.46 ± 1.07	0.35	0.10; 0.60	0.79 ± 0.49	0.99 ± 0.43	0.20	−0.34; 0.74	0.25
**Perceived symptoms after loaded breathing**									
Perceived dyspnea, MBS, /10	4 ± 2	3 ± 2	−1	−2; 0	5 ± 2	4 ± 2	−1	−2; 0	0.65
Perceived respiratory effort, MBS, /10	5 ± 2	4 ± 2	−1	−2; 0*	5 ± 2	4 ± 1	−1	−2; 0	0.59
Unpleasantness, VAS, /10	4 ± 2	3 ± 3	−1	−3; 0	4 ± 3	3 ± 1	−1	−2; 0	0.89

Patients in both groups performed a loaded breathing task at the initiation (pre) and the end (post) of the inspiratory muscle training period. Data of posttest missing for two patients in the control group. Hemodynamic and respiratory responses to the loaded breathing task are expressed as the difference between rest value before the initiation of the loaded breathing task and the value at the end of the loaded breathing task. Data of the pre and post measurement are presented with the difference between the post measurement and the pre measurement. Arterial oxygen saturation was measured by a pulse oximetry. IMT, inspiratory muscle training; SD, standard deviation; CI, confidence interval; MAP, mean arterial blood pressure; RR, respiratory rate; Pinsp., inspiratory pressure; insp., inspiratory; Vt, tidal volume; FVC, forced vital capacity; WoB, work of breathing; PoB, power of breathing; MBS, modified BORG scale; VAS, visual analog scale. *Significance level: p-value < 0.05.

### Inspiratory Muscle Oxygenation During the Standardized Loaded Breathing Task

Table 5 presents the changes in scalene and sternocleidomastoid muscle oxygenation parameters for both groups. In the intervention group, the increase during the standardized loaded breathing task in scalene muscle, oxy (Hb + Mb), deoxy (Hb + Mb), and total (Hb + Mb) from rest were significantly greater at post compared to pre measurements. Additionally, in the intervention group, the decrease in nadir scalene muscles %StiO_2_ was less at post measurements compared to pre measurements (*p* = 0.05). In the control group, no significant changes at post measurements were observed in scalene muscles oxygenation parameters. Furthermore, scalene muscle %StiO_2nadir_ normalized for the mean PoB significantly improved in both groups. No between-group significant differences were observed for all scalene muscle oxygenation parameters.

**TABLE 5:** Changes in inspiratory muscles oxygenation parameters during the standardized loaded breathing tasks.

	Intervention group *n* = 22				Control group *n* = 19				Between group comparison
			
	Pre	Post	Estimated mean difference (post–pre)		Pre	Post	Estimated mean difference (post–pre)		Multivariate effect
	Mean *SD*	Mean *SD*	Mean	95%CI	Mean *SD*	Mean *SD*	Mean	95%CI	*p*-value
**Changes in scalene muscle oxygenation parameters**									
**Δ** Oxygenated [Hb + Mb], μ mol/L	1.9 ± 5.5	6.6 ± 5.4	+4.7	1,6; 7,9*	4.0 ± 4.0	5.6 ± 5.0	+1.7	−1.8; 5.2	0.28
**Δ** Deoxygenated [Hb + Mb], μ mol/L	2.8 ± 3.9	6.5 ± 6.4	+3.7	0.7; 6.7*	4.7 ± 4.1	5.3 ± 3.9	+0.7	−2.7; 4.0	0.22
**Δ** Total [Hb + Mb], μ mol/L	4.6 ± 9.2	13.0 ± 11.1	+8.4	2.5; 14.2*	8.4 ± 7.6	10.9 ± 8.6	+2.5	−4.0; 9.0	0.24
**Δ** Stio_2,_ %	−3.2 ± 2.4	−1.6 ± 1.9	+1.5	−0.3; 3.3	−3.5 ± 3.7	−3.1 ± 3.6	+0.4	−1.6; 2.5	0.30
**Δ** Stio_2 nadir,_ %	−4.0 ± 3.5	−2.3 ± 1.8	+1.8	0.1; 3.5*	−4.1 ± 2.4	−3.5 ± 2.8	+0.6	−1.3; 2.5	0.30
**Δ** Stio_2 nadir_/mean PoB, %/watt	−15.1 ± 13.8	−6.9 ± 6.4	+8.3	0.9; 15.6*	−18.4 ± 15.4	−10.2 ± 8.2	+8.2	0.1; 16.3*	0.41
**Changes in sternocleidomastoid muscle oxygenation parameters**									
**Δ** Oxygenated [Hb + Mb], μ mol/L	2.8 ± 4.6	2.9 ± 3.3	+0.1	−2.8; 3.0	3.6 ± 5.9	3.6 ± 4.8	+0.0	−3.3; 3.4	0.73
**Δ** Deoxygenated [Hb + Mb], μ mol/L	4.2 ± 3.9	3.5 ± 3.7	−0.8	−3.2; 1.6	4.6 ± 4.4	4.5 ± 3.2	−0.2	−2.9; 2.6	0.67
**Δ** Total [Hb + Mb], μ mol/L	7.1 ± 6.9	6.4 ± 6.3	−0.7	−5.4; 4.0	8.1 ± 9.7	8.1 ± 7.4	0.0	−5.4; 5.5	0.72
**Δ** Stio_2,_ %	−4.4 ± 4.2	−2.5 ± 3.0	+1.8	−0.6; 4.3	−4.5 ± 4.0	−5.2 ± 4.3	−0.7	−3.5; 2.2	0.09
**Δ** Stio_2 nadir,_ %	−5.7 ± 6.1	−3.4 ± 3.2	+2.3	−0.6; 5.2	−5.7 ± 4.5	−6.7 ± 4.1	−1.0	−4.3; 2.4	0.02
**Δ** Stio_2 nadir_/mean PoB, %/watt	−27.9 ± 55.2	−9.6 ± 12.1	+18.4	−1.4; 38.1	−21.0 ± 13.2	−17.3 ± 10.4	+3.7	−18.7; 26.0	< 0.01

Changes from rest of scalene and sternocleidomastoid muscles in oxygenation parameters during the standard loaded breathing task at the initiation (pre) and end (post) of the inspiratory muscle training period. Data of posttest missing in one patient in the intervention group and two patients in the control group. Oxygenated [Hb + Mb], oxygenated hemoglobin and myoglobin; deoxygenated [Hb + Mb], deoxygenated hemoglobin and myoglobin; total [Hb + Mb], total hemoglobin and myoglobin; StiO_2_, muscle oxygen saturation index; SD, standard deviation. CI, confidence interval. *Significance level: p-value < 0.05.

For the sternocleidomastoid muscle, neither within group nor between-group significant differences were found for changes in oxy (Hb + Mb), deoxy (Hb + Mb), and total (Hb + Mb) concentration. However, a significant smaller decrease in nadir %StiO_2_ following the IMT period was observed in the high-intensity IMT compared to the sham low-intensity IMT group (Table 5). In addition, at post measurement the sternocleidomastoid muscle %StiO_2nadir_ normalized for the mean PoB was significantly more improved in the high-intensity IMT compared to the sham low-intensity IMT (Table 5).

### Verifying for Potential Confounders

The apparent baseline differences between the groups in age and FVC % predicted at baseline were considered as potential confounder of the treatment effect on the primary outcome (Table 1 and Supplementary Table 2). Weak and non-significant correlations between the baseline variables and change in muscle oxygen saturation index (age: *r* = −0.24, FVC % predicted: *r* = −0.11, Supplementary Table 2) and muscle oxygen saturation index normalized for the power output (age: *r* = 0.15, FVC % predicted: *r* = 0.27, Supplementary Table 2) for the scalene muscle were observed. Similarly, weak and non-significant correlations were observed between the baseline variables and change in muscle oxygen saturation index (age: *r* = −0.11, FVC % predicted: *r* = −0.17, Supplementary Table 2) and muscle oxygen saturation index normalized for the power output (age: *r* = 0.02, FVC % predicted: *r* = −0.18, Supplementary Table 2) for the sternocleidomastoid muscle. Therefore, age and FVC % predicted could be disregarded as confounding factors of the treatment effect on the oxygenation variables.

## Discussion

### Main Findings

The aim of the present study was to compare the effects of IMT on scalene and sternocleidomastoid inspiratory muscle oxygenation parameters during a standardized loaded breathing tasks between patients with weaning difficulties who received high-intensity IMT (intervention group) or low-intensity IMT (control group). The main findings of the study are as follows: (1) scalene muscle oxygen delivery [oxy (Hb + Mb)], oxygen utilization [deoxy (Hb + Mb)], blood volume [total (Hb + Mb)], and oxygen saturation index (StiO_2_%_nadir_) improved significantly in the intervention group after high-intensity IMT, potentially to support the greater PoB during the standardized loaded breathing task. In the control group, scalene muscle oxygen delivery, utilization, blood volume and oxygen saturation index improved to a lesser but non-significant extend after low-intensity IMT. We did not observe statistical significance differences in the magnitude of improvements in the aforementioned oxygenation parameters between the intervention and control group. Furthermore, scalene muscle %StiO_2_ normalized for the power output improved with a similar magnitude in both groups. (2) Following the IMT period, the intervention group exhibited a significantly greater improvement in sternocleidomastoid muscle StiO_2_%_nadir_ and StiO_2_%_nadir_ normalized for the power output compared to the control group. (3) Respiratory muscle strength improved in both groups. However, only in the intervention group FVC and peak inspiratory flow improved. Collectively these findings suggest that high-intensity IMT induces greater improvements in scalene and sternocleidomastoid inspiratory muscle oxygenation parameters and respiratory muscle function, FVC and PIF, compared to low-intensity IMT in patients with weaning difficulties.

### Adaptation Patterns of Scalene and Sternocleidomastoid Inspiratory Muscle Oxygenation Parameters in Response to Inspiratory Muscle Training

To our knowledge, this is the first study that explored the effect of IMT on the oxygenation parameters of the scalene and sternocleidomastoid inspiratory muscles in patients with weaning difficulties. Different patterns of adaptations, as a response to the different intensity of muscle stimuli, were observed between the scalene and sternocleidomastoid muscles. The different adaptation patterns of changes in oxygenation parameters after high-intensity or sham low-intensity IMT can be attributed to differences in recruitment and activation properties of the muscles. The function of the scalene and sternocleidomastoid muscles are similar, cranial displacement of the sternum and ribcage, nevertheless the recruitment pattern of the muscles is very different (Hudson et al., 2007). The scalene muscles, are primary inspiratory muscles and are activated from the onset of the inspiration during tidal breathing, static maximal inspirations (PImax) and dynamic inspirations up to total lung capacity (FVC measurement) (Campbell, 1955; Hudson et al., 2007). In contrast, the sternocleidomastoid muscle is an accessory muscle and is not activated during tidal breathing and the onset of activation is later during static (PImax) and dynamic inspirations (FVC) (Hudson et al., 2007). Additionally, the activation of the sternocleidomastoid muscle has been shown to be larger than the activation of the diaphragm, scalene or intercostal muscles and increases progressively during the static and dynamic inspirations (Hudson et al., 2007). The greater activation of the sternocleidomastoid muscle during increased ventilatory demands is attributed to its length-tension relationship at higher lung volumes, which results in a lesser mechanical advantage relative to the parasternal intercostal and the scalene muscles (Basoudan et al., 2020; Derbakova et al., 2020). Thus the sternocleidomastoid muscle shortens more for a given change in pleural pressure than the intercostal muscles or scalene muscles (Hudson et al., 2007). In addition, the greater activation of the sternocleidomastoid muscle can be as well attributed to its ability to generate stronger and faster contractions compared to other inspiratory muscles such as the scalene muscle and diaphragm (De Troyer and Boriek, 2011; Basoudan et al., 2020). The preferable strategy of the inspiratory muscles is to rely on the increase in activation of the accessory inspiratory muscles (i.e., sternocleidomastoid muscle) in response to increased ventilator demands. The increased load imposed by IMT is shared by the accessory and primary inspiratory muscles and protects against the development of respiratory muscle fatigue (Basoudan et al., 2020). The different recruitment patterns between the scalene muscle and the sternocleidomastoid muscle during IMT might support the different oxygenation patterns observed in the present study.

Due to the early recruitment of the scalene muscles, these muscles may receive a comparable training stimulus with both high-intensity IMT (intervention group) and low-intensity IMT (control group) to induce similar improvements in oxygenation parameters between the two groups. In contrast, the stronger contraction of the sternocleidomastoid muscle during high compared to low-intensity IMT may result in greater development of intramuscular pressures thus inhibiting its perfusion during repetitive dynamic contractions (De Troyer et al., 1994; De Troyer and Boriek, 2011). However, only high-intensity IMT was able to induce a lesser decrease in muscle oxygen saturation index of the sternocleidomastoid muscle and a lesser decrease in muscle oxygen saturation index normalized by the power output following the training period compared to low intensity IMT. The latter finding may suggests that the sternocleidomastoid muscle had an improved muscle efficiency following high-intensity IMT. Indeed, muscle efficiency is the ability of a patient to convert energy consumed into external power which is a key determinant for exercise performance (Böning et al., 2017). In muscular exercise, muscle efficiency is calculated as the ratio of mechanical power output to the total metabolic cost (Edwards, 1978; Böning et al., 2017). In the present study, assuming that the oxygen cost of inspiratory muscles can be represented by the changes in oxygen saturation index (%StiO_2_), our findings may suggests that high-intensity IMT can decrease the oxygen cost of scalene muscles and to a greater extent of sternocleidomastoid muscle for a given power output compared to low intensity IMT.

### Breathing Pattern and Respiratory Muscle Function in Response to Inspiratory Muscle Training

Patients of the intervention group initiated IMT with an external load of 34 ± 5% PImax and could increase the load to almost the double of the initial load, 62 ± 22% PImax measured at baseline. Even though the training load doubled, the patients in the intervention group were able to maintain similar inspiratory flow rates over the course of the IMT period and even increase the tidal volume. Similar responses in breathing characteristics were observed in the control group. As the volume responses and flow responses were similar between the groups but the training load was significantly higher in the intervention group, this resulted in significant higher WoB and PoB performed during the training. In COPD patients higher WoB and PoB generated by the inspiratory muscles, is related to improvements in inspiratory muscle strength and endurance after IMT (Charususin et al., 2018). Remarkably, inspiratory muscle strength improved similarly in the intervention and control group. In comparison to findings in previous studies on IMT in mechanically ventilated patients (PImax: +7 cmH_2_O) the increase in PImax in both groups was considerable larger in the present study (Elkins and Dentice, 2015). This indicate that specifically patients with weaning difficulties have higher potential to benefit from IMT. In addition, the lack of difference in improvements on PImax between the groups may indicate that the sham low-intensity IMT protocol might introduced a sufficient training stimulus to improve inspiratory muscle strength in patients with weaning difficulties. This statement is supported by the improved muscle oxygen saturation index of the scalene muscles in both groups and the surprising finding that patients in the control group indicated moderate to high symptoms scores after IMT, which were equally high as the perceived symptoms the patients of the intervention group reported. Nevertheless, high-intensity IMT showed superior responses in other respiratory muscle function parameters such as FVC and PIF compared to sham low-intensity. Only the intervention group, improved FVC and PIF. It seems that high-intensity IMT training has the capacity to improve overall respiratory muscle function while sham low-intensity IMT can influence inspiratory muscle strength. An ongoing larger randomized controlled trial will provide tangible evidence on the effect of IMT on the respiratory muscle function and weaning outcomes (Hoffman et al., 2018).

### Strengths, Methodological Considerations and Study Limitations

An important strength of the present study is the use of NIRS, as a non-invasive method for simultaneously assessing local inspiratory muscles oxygenation parameters in the challenging environment of intensive care. This novel concept could potentially be the first step toward utilization of this technology for assessing the effectiveness of different types of exercise interventions on critically ill patients. Another strength of the study is the stratification of a control group (low-intensity IMT) which allowed better appreciating the effects of high-intensity inspiratory muscles training stimulus in patients with weaning difficulties.

The approach of the statistical inference through estimation might also provide a different point of view (Elkins et al., 2021). Muscle oxygen delivery utilization, blood volume and oxygen saturation index of the scalene, FVC and PIF improved significantly only after high-intensity IMT. However, this was not translated into a statistical significant difference between the intervention and the control group. It is reasonable that due to the relatively small sample size and considerable variability, the present study was underpowered to identify statistical significant differences with null hypothesis statistical test for changes in oxygenation parameters of the scalene and respiratory muscle function between the intervention and control group. It has been recently recommended to use estimation methods as a statistical inference approach. Nevertheless, the observed treatment effect sizes (estimated mean difference pre–post treatment with 95%CI, Tables 3, 5) of the scalene muscle oxygenation parameters, FVC and PIF suggest larger improvements in response to high-intensity IMT compared to the response during low intensity-IMT. However, due to the wide confidence intervals of similar size of these variables in both groups, the treatment effect is to imprecise to conclude with certainty that the observed difference will be observed in clinical practice (Amrhein et al., 2019; Elkins et al., 2021). In addition, interpretation of the estimates of the treatment effects is rendered more difficult due to the lack of an established minimal clinically important difference for these variables (Ferreira, 2018; Elkins et al., 2021).

The effect on PImax and inspiratory muscle oxygen saturation index normalized for the power output of the scalene in the control group, may be the result of the following methodological considerations and/or limitations of the present study. Methodologically, the improvements of respiratory muscle function could be attributed to the placebo effect (Hróbjartsson and Gøtzsche, 2010). Alternatively, the single standardized loaded breathing task performed at the beginning of the IMT period with a load corresponding to 30% of the PImax could have induced a training effect in the control group. However, the possibility that a single loaded breathing task inflicts a training effect is nihil as it is indicated that a training effect can be expected after approximately 16 sessions (Kraemer et al., 1996). Furthermore, the sham low-intensity IMT protocol imposed an external load on the inspiratory muscles, which is in fact very small. However, the current study design did not allow measurement of the lung and chest wall resistance the patient had to overcome during the training sessions. Therefore, the total WoB and PoB that the patients had to generate during the IMT sessions is unknown. Subsequently, it is possible that the load imposed on the inspiratory muscles was high enough to impose a training stimulus in the low-intensity IMT group. Future studies should assess changes in esophageal pressures and transdiaphragmatic pressures during IMT to measure the needed changes in pleural pressure to generate enough WoB and PoB to overcome the internal and external resistance. Pleural pressures cannot be measured directly at the bedside, but are best reflected by the esophageal pressure measurements (Pasticci et al., 2020). Furthermore, all patients received a comprehensive usual care protocol comprising of early mobilization, sedation protocol and weaning protocol. These background interventions have been proven to have beneficial effects on the weaning and respiratory muscle strength (Smuder et al., 2012; Worraphan et al., 2020). Therefore, a larger sample size will be needed to identify the possible additional effect of IMT on top of the usual care package (Hoffman et al., 2018). To identify the underlying causes of the similar improvement of PImax following high-intensity or low-intensity IMT, future studies need to focus on the possible effects of low-intensity IMT vs. high-intensity IMT and include a usual care control group.

Performing clinical trials in an intensive care setting can be challenging, as patient’s clinical condition can deteriorate rapidly. Therefore, we aimed to train the patient daily to increase the amount of possible training days with the knowledge that the clinical condition could prevent IMT on particular days. Despite the challenging setting, an equal and high completion rate of IMT sessions was obtained in both groups. A limitation of the standardized loaded breathing task was the inability to standardize the breathing pattern as it was not feasible in the intensive care setting. Due to the higher tidal volume and inspiratory flow in the post standardized loaded breathing task the WoB and PoB could not be standardized. The effect on the muscle oxygen saturation index of the inspiratory muscles has been masked by the increased power output. Therefore, data was provided on the oxygen saturation index normalized for the power output (Barroco et al., 2017; Gephine et al., 2021).

The influence of possible selection bias was limited by performing a MANOVA, as this type of analyses will take in to account missing values at the pre or post measurement. However, the standardized loaded breathing task, evaluated with NIRS, could not be executed in one patient in both groups due to death or successful weaning after familiarization with IMT (after 1 or 2 IMT sessions, Figure 3). Three patients were excluded from the analyses due to insufficient quality of the NIRS signal and one patient was completely random missing because of an error in the protocol, an inappropriate low load (<10% PImax) was imposed during the standardized loaded breathing task.

### Clinical Implications and Future Considerations

Weakness of the inspiratory muscles may compromise spontaneous breathing (Parthasarathy et al., 2007; De Troyer and Boriek, 2011). By improving the oxygenation parameters of inspiratory muscles, high-intensity IMT may contribute to the prevention of respiratory muscle fatigue (Tobin et al., 2012; Rodrigues et al., 2019; Basoudan et al., 2020) during spontaneous breathing trial. Respiratory muscle fatigue is considered a key contributor to weaning failure (Parthasarathy et al., 2007). Whether potential improvements in inspiratory muscle oxygenation parameters following IMT are associated with better weaning outcomes, is of specific interest to be investigated in critically ill patients with weaning difficulties. The effect of high-intensity IMT versus sham low-intensity IMT on clinical outcomes will be reported in an ongoing clinical trial (Hoffman et al., 2018).

Prolonged mechanical ventilation decreases the ability of the inspiratory muscles to augment the blood flow to match the oxygen demand in response to the higher contractile activity during spontaneous breathing (Davis et al., 2012; Duan and Bai, 2020). Furthermore, skeletal muscle fatigue is associated with less capillary density (Tickle et al., 2020). It is possible that after high-intensity IMT, the improvements in scalene and sternocleidomastoid muscles oxygenation profile could be due to structural adaptations such as improvements in type I fiber proportion, capillary density and oxygen delivery that in turn might contribute to the increased exercise tolerance (higher PoB and WoB) (Kraemer et al., 1996; Bottinelli and Reggiani, 2000; Ramirez-Sarmiento et al., 2002). Nevertheless, as this constitutes an exploratory study the above-mentioned causalities are potential hypotheses for future studies. Therefore, it is warranted to evaluate the muscle properties by performing micro-biopsies of the extradiaphragmatic inspiratory muscles in future studies. Furthermore, neuromuscular adaptations of the motor unit synchronization could have occurred following high-intensity IMT (Gabriel et al., 2006). It should be investigated whether motor units of the inspiratory muscles are able to fire faster and more powerful, and subsequently influencing the intramuscular pressure by shortening the muscle contraction time (Gabriel et al., 2006). Hence, respiratory muscle function should be comprehensively evaluated with both non-invasive (i.e., surface EMG of primary and accessory respiratory muscles and ultrasound evaluation of respiratory muscle thickness and thickening fraction) and invasive (esophageal, abdominal and transdiaphragmatic pressures, and EMG of the diaphragm) measurements.

Finally, future studies should investigate the optimization of the IMT stimulus in patients with weaning difficulties. The effect of high-intensity IMT and low-intensity IMT compared to a usual care group should be further investigated as it seems from the present study that low-intensity IMT resulted in benefits on scalene muscle oxygen saturation index in patients with weaning difficulties.

## Conclusion

The results of this exploratory study suggest similar improvements of scalene muscle oxygen saturation index and inspiratory muscle strength following high and low-intensity IMT in patients with weaning difficulties. However, larger improvements in sternocleidomastoid muscle oxygen saturation index and FVC and peak inspiratory flow following high-intensity IMT were observed.

Further research is warranted to investigate the physiological mechanisms behind these improvements in the extradiaphragmatic respiratory muscles oxygenation (i.e., structural and neuromuscular) following IMT as well as whether these beneficial effects of high-intensity versus low-intensity IMT can be translated into better weaning outcomes in critically ill patients with weaning difficulties.

## Data Availability Statement

The original contributions presented in the study are included in the article/Supplementary Material, further inquiries can be directed to the corresponding author.

## Ethics Statement

The studies involving human participants were reviewed and approved by Ethische Commissie Onderzoek UZ/KU Leuven. The patients (if awake and alert) or a family member provided their written informed consent to participate in this study.

## Author Contributions

ZL, GH, JM, RG, DL, and MV contributed to conception and design of the study. ZL, DP, BC, and MV performed the data collection. MV and DP organized the database. MV performed the statistical analysis, and wrote the first draft of the manuscript. All authors contributed to manuscript revision, read, and approved the submitted version.

## Conflict of Interest

The authors declare that the research was conducted in the absence of any commercial or financial relationships that could be construed as a potential conflict of interest.

## Publisher’s Note

All claims expressed in this article are solely those of the authors and do not necessarily represent those of their affiliated organizations, or those of the publisher, the editors and the reviewers. Any product that may be evaluated in this article, or claim that may be made by its manufacturer, is not guaranteed or endorsed by the publisher.

## Abbreviations

IMT: inspiratory muscle training
PImax: maximal inspiratory pressure
PoB: power of breathing
Δ %StiO_2_: muscle oxygen saturation index
ICU: intensive care unit
EMG: electromyographic
NIRS: near-infrared spectroscopy
COPD: chronic obstructive pulmonary disease
APACHE II: Acute Physiology and Chronic Health Evaluation II Scale
WoB: work of breathing
FVC: forced vital capacity
PIF: peak inspiratory flow
Hb: hemoglobin
Mb: myoglobin
MAP: mean arterial blood pressure.
