# Supplementary material for: High-Intensity Inspiratory Muscle Training Improves Scalene and Sternocleidomastoid Muscle Oxygenation Parameters in Patients With Weaning Difficulties: A Randomized Controlled Trial
Source: Front Physiol. 2022 Feb 9;13:786575. doi: 10.3389/fphys.2022.786575 (PMC8864155; doi:10.3389/fphys.2022.786575)
Supplement: Supplementary file 1 [file Table_1.docx]

| **Table S1 hemodynamic and respiratory parameters at rest** | | | | | | | | | | | | | | | | | | | | |
| --- | --- | --- | --- | --- | --- | --- | --- | --- | --- | --- | --- | --- | --- | --- | --- | --- | --- | --- | --- | --- |
|  | **Intervention group n= 21** | | | | | | | | | **Control group n= 17** | | | | | | | | | | |
|  | **Pre** | | | **Post** | | | **Difference**  **Post-Pre** | | | **Pre** | | | **Post** | | | **Difference**  **Post-Pre** | | | **Interaction effect**  **(group x time)** | |
|  | Mean |  | SD | Mean |  | SD | Mean |  | SD | Mean |  | SD | Mean |  | SD | Mean |  | SD | | ***p-value*** |
| **Heart rate at rest , beats/min** | 93 | ± | 21 | 85 | ± | 16 | **-8** | **±** | **23** | 89 | ± | 21 | 88 | ± | 19 | **-1** | **±** | **19** | *0.35* | |
| **MAP, mmHg** | 82 | ± | 15 | 84 | ± | 12 | **3** | **±** | **15** | 81 | ± | 7 | 80 | ± | 13 | **-1** | **±** | **14** | *0.59* | |
| **Respiratory rate, breaths/min** | 24 | ± | 6 | 23 | ± | 4 | **-1** | **±** | **8** | 20 | ± | 4 | 21 | ± | 4 | **1** | **±** | **6** | *0.49* | |
| **SpO_2,_ %** | 98 | ± | 3 | 97 | ± | 3 | **-1** | **±** | **4** | 98 | ± | 2 | 97 | ± | 3 | **-1** | **±** | **3** | *0.94* | |
| **Haemoglobin, ml/dl** | 8.5 | ± | 1.1 | 8.9 | ± | 1.1 | **0.4** | **±** | **1.1** | 8.7 | ± | 0.7 | 8.7 | ± | 0.9 | **0.04** | **±** | **1** | *0.32* | |
| **PaO_2_, mmHg** | 84.4 | ± | 13.8 | 83.7 | ± | 15.7 | **-0.7** | **±** | **20.2** | 78.1 | ± | 14.0 | 83.3 | ± | 15.2 | **5.2** | **±** | **15.1** | *0.64* | |
| **SaO_2_, %** | 97.0 | ± | 1.5 | 96.6 | ± | 1.8 | **-0.3** | **±** | **1.9** | 95.7 | ± | 3.3 | 96.3 | ± | 3.2 | **0.6** | **±** | **2.6** | *0.34* | |
| **Fraction of Inspired O_2_, %** | 0.29 | ± | 0.06 | 0.30 | ± | 0.06 | **0.01** | **±** | **0.02** | 0.35 | ± | 0.10 | 0.34 | ± | 0.10 | **-0.01** | **±** | **0.02** | *0.07* | |
