# Supplementary material for: High-Intensity Inspiratory Muscle Training Improves Scalene and Sternocleidomastoid Muscle Oxygenation Parameters in Patients With Weaning Difficulties: A Randomized Controlled Trial
Source: Front Physiol. 2022 Feb 9;13:786575. doi: 10.3389/fphys.2022.786575 (PMC8864155; doi:10.3389/fphys.2022.786575)
Supplement: Supplementary file 2 [file Table_2.docx]

| **Table S2: Covariate analyses** | | | | | | | | | | | |
| --- | --- | --- | --- | --- | --- | --- | --- | --- | --- | --- | --- |
|  |  | **∆ StiO_2 nadir_**  **Scalene muscle** | | | | | **∆ StiO_2 nadir_**  **Sternocleidomastoid muscle** | | | | |
| **descriptive analysis** |  | **Mean** | **SD** | | | | **Mean** | **SD** | | | |
|  | **Control group** | 0.6 | 2.9 | | | | -1.0 | 5.3 | | | |
|  | **Intervention group** | 1.8 | 3.7 | | | | 2.3 | 5.0 | | | |
| **Independent covariate analyses** | |  |  | |  | |  |  | | |  |
| **Age** | | | | | | | | | | | |
| **Correlation** |  | **r** | | **p-value** | | | **r** | | **p-value** | | |
|  |  | -0.24 | | 0.14 | | | -0.11 | | 0.51 | | |
| **ANCOVA analysis** |  | **Mean Square** | **F** | | **p-value** | | **Mean Square** | **F** | | | **p-value** |
| **Tests of between subjects Effects, Source** | **Group** | 32.8 | 3.1 | | 0.09 | | 39.3 | 1.5 | | | 0.23 |
|  | **Age** | 49.7 | 4.7 | | 0.07* | | 32.6 | 1.3 | | | 0.27 |
| **Estimates** |  | **Mean** | **SE** | | | | **Mean** | **SE** | | | |
| Covariate appearing in the model are evaluated at:  Age= 57 years | **Control group** | 2.7 | 1.3 | | | | 1.3 | 2.1 | | | |
|  | **Intervention group** | 1.7 | 0.8 | | | | 2.6 | 1.2 | | | |
|  |  |  |  | | | |  |  | | | |
| **FVC_baseline_, %pred** | | | | | | | | | | | |
| **Correlation** |  | **r** | | **p-value** | | | **r** | | **p-value** | | |
|  |  | -0.11 | | 0.50 | | | -0.17 | | 0.31 | | |
| **ANCOVA analysis** |  | **Mean Square** | **F** | | | **p-value** | **Mean Square** | **F** | | **p-value** | |
| **Tests of between subjects Effects, Source** | **Group** | 1.6 | 0.1 | | | 0.72 | 19.9 | 0.7 | | 0.40 | |
|  | **FVC_baseline_, %pred** | 0.13 | 0.0 | | | 0.92 | 4.3 | 1.6 | | 0.70 | |
| **Estimates** |  | **Mean** | **SE** | | | | **Mean** | **SE** | | | |
| Covariate appearing in the model are evaluated at:  FVC_baseline_, %pred = 26% | **Control group** | 0.8 | 0.9 | | | | -0.9 | 1.4 | | | |
|  | **Intervention group** | 2.0 | 0.8 | | | | 2.1 | 1.3 | | | |
|  | | **∆ post-pre Stio_2 nadir_/ mean PoB Scalene muscle** | | | | | **∆ post-pre Stio_2 nadir_/ mean PoB Sternocleidomastoid muscle** | | | | |
| **descriptive analysis** |  | **Mean** | **SD** | | | | **Mean** | **SD** | | | |
|  | **Control group** | 8.2 | 13.3 | | | | 4.1 | 13.2 | | | |
|  | **Intervention group** | 8.3 | 12.0 | | | | 18.1 | 44.1 | | | |
| **Independent covariate analyses** | |  |  | |  | |  |  | | |  |
| **Age** | | | | | | | | | | | |
| **Correlation** |  | **r** | | **p-value** | | | **r** | | **p-value** | | |
|  |  | r= 0.15 | | 0.38 | | | 0.02 | | 0.89 | | |
| **ANCOVA analysis** |  | **Mean Square** | **F** | | **p-value** | | **Mean Square** | **F** | | | **p-value** |
| **Tests of between subjects Effects, Source** | **Group** | 74.2 | 0.5 | | 0.50 | | 3.0 | 0.0 | | | 0.96 |
|  | **Age** | 188.1 | 1.2 | | 0.29 | | 74.5 | 0.1 | | | 0.80 |
| **Estimates** |  | **Mean** | **SE** | | | | **Mean** | **SE** | | | |
| Covariate appearing in the model are evaluated at:  Age= 57 years | **Control group** | 4.7 | 5.0 | | | | 4.2 | 11.4 | | | |
|  | **Intervention group** | 9.1 | 3.0 | | | | 20.0 | 7.9 | | | |
|  |  |  |  | | | |  |  | | | |
| **FVC_baseline_, %pred** | | | | | | | | | | | |
| **Correlation** |  | **r** | | **p-value** | | | **r** | | **p-value** | | |
|  |  | 0.27 | | 0.10 | | | -0.18 | | 0.28 | | |
| **ANCOVA analysis** |  | **Mean Square** | **F** | | | **p-value** | **Mean Square** | **F** | | **p-value** | |
| **Tests of between subjects Effects, Source** | **Group** | 135.8 | 0.9 | | | 0.35 | 4673.6 | 4.3 | | **0.04*** | |
|  | **FVC_baseline_, %pred** | 219.3 | 1.4 | | | 0.24 | 2256.1 | 2.1 | | 0.16 | |
| **Estimates** |  | **Mean** | **SE** | | | | **Mean** | **SE** | | | |
| Covariate appearing in the model are evaluated at:  FVC_baseline_, %pred = 26% | **Control group** | 6.2 | 3.2 | | | | 2.8 | 8.3 | | | |
|  | **Intervention group** | 8.7 | 3.1 | | | | 9.3 | 8.5 | | | |
| Δ Stio2 nadir/ mean PoB: Oxygen extraction normalized for the power output was calculated as the changes from rest in oxygenation in the muscle per unit of power of breathing performed during loaded breathing, SD: standard deviation, SE: standard error, ANCOVA: Analysis of covariance, FVC: forced vital capacity, %pred: percentage of the predicted value. Significance level: p-value<0.05 | | | | | | | | | | | |
